# Extracorporeal membrane oxygenation technology for adults: an evidence mapping based on systematic reviews

**DOI:** 10.1186/s40001-024-01837-0

**Published:** 2024-04-22

**Authors:** Kai Xie, Hui Jing, Shengnan Guan, Xinxin Kong, Wenshuai Ji, Chen Du, Mingyan Jia, Haifeng Wang

**Affiliations:** 1 Department of Respiratory Medicine, The First Affiliated Hospital of Henan University of Chinese Medicine, Zhengzhou, China; 2 Academy of Chinese Medical Sciences, Henan University of Chinese Medicine, Zhengzhou, China; 3 Co-construction Collaborative Innovation Center for Chinese Medicine and Respiratory Diseases by Henan & Education Ministry of People’s Republic of China, Henan University of Chinese Medicine, Zhengzhou, China

**Keywords:** Extracorporeal membrane oxygenation, Evidence mapping, Human health, Systematic review

## Abstract

**Background:**

Extracorporeal membrane oxygenation (ECMO) is a cutting-edge life-support measure for patients with severe cardiac and pulmonary illnesses. Although there are several systematic reviews (SRs) about ECMO, it remains to be seen how quality they are and how efficacy and safe the information about ECMO they describe is in these SRs. Therefore, performing an overview of available SRs concerning ECMO is crucial.

**Methods:**

We searched four electronic databases from inception to January 2023 to identify SRs with or without meta-analyses. The Assessment of Multiple Systematic Reviews 2 (AMSTAR-2) tool, and the Grading of Recommendations Assessment, Development and Evaluation (GRADE) system were used to assess the methodological quality, and evidence quality for SRs, respectively. A bubble plot was used to visually display clinical topics, literature size, number of SRs, evidence quality, and an overall estimate of efficacy.

**Results:**

A total of 17 SRs met eligibility criteria, which were combined into 9 different clinical topics. The methodological quality of the included SRs in this mapping was “Critically low” to “Moderate”. One of the SRs was high-quality evidence, three on moderate, three on low, and two on very low-quality evidence. The most prevalent study used to evaluate ECMO technology was observational or cohort study with frequently small sample sizes. ECMO has been proven beneficial for severe ARDS and ALI due to the H1N1 influenza infection. For ARDS, ALF or ACLF, and cardiac arrest were concluded to be probably beneficial. For dependent ARDS, ARF, ARF due to the H1N1 influenza pandemic, and cardiac arrest of cardiac origin came to an inconclusive conclusion. There was no evidence for a harmful association between ECMO and the range of clinical topics.

**Conclusions:**

There is limited available evidence for ECMO that large sample, multi-center, and multinational RCTs are needed. Most clinical topics are reported as beneficial or probably beneficial of SRs for ECMO. Evidence mapping is a valuable and reliable methodology to identify and present the existing evidence about therapeutic interventions.

## Introduction

Extracorporeal membrane oxygenation (ECMO) is an advanced life-support technique to rescue critically ill patients with the severe cardiac and pulmonary disease [1]. In 1970, Robert et al. provided a patient with adult respiratory distress syndrome (ARDS) with three days of cardiopulmonary support, establishing a record for long-term life support [2]. Nonetheless, two randomized controlled trials (RCTs) on the clinical application concluded in 1979 [3] and 1994 [4] concluded that ECMO did not increase the likelihood of survival in patients with severe acute respiratory failure (ARF) or ARDS. This conclusion excluded ECMO, primarily used to treat cardiopulmonary failure in infants. Until 2009, the Lancet published the third RCT study result of ECMO technology for ARDS [5], which revealed that 63% of the ECMO group survived to 6 months without disability, 16% more than the conventional management group. In the same year, 2009, a clinical study [6] on treating the influenza A (H1N1)-associated ARDS published in the JAMA demonstrated that approximately one-third of mechanically ventilated patients treated with ECMO had a 79% survival rate. However, the results of EOLIA (ECMO to Rescue Lung Injury in Severe ARDS) in 2018 showed that 60-day mortality was not significantly lower with ECMO than with a strategy of conventional mechanical ventilation (MV) that included ECMO as rescue therapy among patients with very severe ARDS. There is still significant controversy over the effectiveness of ECMO.

Due to technological advancements, improved safety, and reduced complications [7], especially in the context of the coronavirus disease 2019 (COVID-19) pandemic, there is a growing demand for treatment techniques for ECMO. According to the Extracorporeal Life Support Organization (ELSO) [8], an international voluntary registry founded in 1989, the number of ECMO runs has increased by 1137% over the past nearly two decades, from 1643 between 1990 and 2021 to over 21,896 to date. Similarly, the number of ECMO centers, which increased by 125% (from 83 to 187) between 1990 and 2010, rose by a staggering 216% (from 187 to 591) between 2010 and 2021, and reached over 1000 in 2023.

In the era of evidence-based medicine, it is generally accepted that all healthcare decisions should be based on the strongest scientific evidence available [9]. Research on ECMO effects continues to expand, which has been the subject of many primary research studies and systematic reviews (SRs) of the literature. SR, a critical evidence synthesis method, with or without meta-analysis, is widely used in resolving diverse healthcare questions, which are the foundation of evidence-based healthcare provided evidence to support decision-making [10]. In contrast, SR frequently addresses particular concerns, preventing them from providing a comprehensive overview of a given topic [11]. Moreover, the reliability of SR methodological and reporting quality trends to affect the correct evaluation of intervention results [12], and even fragmentary reports may impact on the selection of appropriate intervention measures [13]. Overviews of SRs (or umbrella reviews) attempt to systematically retrieve and summarize the results of multiple SRs into a single document [14]. The number of published overviews of SRs has increased steadily in recent years, in part due to the proliferation of SRs, but methods for conducting, interpreting and reporting overviews are in their infancy [15].

In general, the steps for undertaking an overview mirror those of a systematic review, with many of the methods used in systematic reviews being directly transferrable to overviews (e.g., independent study selection and data extraction) [16]. However, there are unique features of overviews that require the use of different or additional methods, for example, methods for assessing the quality or the risk of bias in systematic reviews, dealing with the inclusion of the same trial in multiple systematic reviews, dealing with out-of-date systematic reviews, and dealing with discordant results across systematic reviews [15]. Evidence maps provide a systematic method for mapping the evidence on a particular topic, which clarifies the characteristics of the studies in this field from multiple dimensions (such as intervention type, research population, research conclusions, etc.), with the resulting map facilitating identification of gaps in the literature, thereby providing decision-makers with systematic evidence support [17, 18]. A key strength of the evidence mapping method is the use of visuals or interactive, online databases.

Increasing numbers of SR for the application of ECMO technology in diseases have been currently conducted, but a comprehensive systematic summary or visual representation of the overall impact of ECMO is lacking, which is where the strength of the evidence map lies. Consequently, we use evidence mapping to identify, characterize, and organize the currently available evidence on ECMO based on the included SRs to provide reliable evidence for ECMO efficacy and safety assessment, as well as guidance for clinical application and future research.

## Methods

### Data sources and search strategy

We searched four electronic databases including Pubmed, EMBASE, Cochrane Library, Web of Science from their inception through January 2023. Search strategies were constructed using combinations of words describing the intervention of interest (“Extracorporeal Membrane Oxygenation” or “Venovenous ECMOs” or “Venovenous Extracorporeal Membrane Oxygenation” or “Venovenous ECMO” or “Venoarterial ECMO” or “Venoarterial ECMOs” or “Venoarterial Extracorporeal Membrane Oxygenation” or “Extracorporeal Membrane Oxygenations” or “ECMO” or “ECMO Treatment” or “ECMO Treatments” or “Extracorporeal Life Support” or “ECMO Extracorporeal Membrane Oxygenation” or “ECLS” or “ECLS Treatment” or “ECLS Treatments” or “Extracorporeal Life Supports” or “Extracorporeal Gas Exchange”), and the studied type (“meta-analysis” or “systematic review”). Depending on characteristics of the database, medical subject headings (MeSH) and free vocabulary words were combined. No language restrictions were imposed.

### Inclusion and exclusion criteria

#### Design

Only SRs with or without meta-analyses on ECMO that compiled primary studies for any clinical indication were eligible for inclusion. We defined SRs as reviews that self-identified as a “systematic review”, “systematic review and meta-analysis”, or “review” and reported the search sources and identified studies. Animal experiments, descriptive studies, conference abstracts, case reports, reviews, clinical experiences, trial protocols, letters, editorials, and unavailable or duplicate publication papers were excluded.

#### Participants

Adult (age ≥ 16 years old) participants with any disease status were included, regardless of gender.

#### Intervention and comparison

SRs describing the effects of ECMO for any clinical indication were eligible for inclusion. SRs were still acceptable if they included other interventions and ECMO results were reported separately. Comparisons were made with conventional treatments, such as conventional MV alone and conventional cardiopulmonary resuscitation (CCPR).

#### Outcomes

SRs presenting clinical topics were included, whereas those focusing on study designs, intervention characteristics, pharmacokinetics, prevalence, prognostic predictors, and cost-effectiveness unrelated to patient clinical topics were excluded.

#### Timing

SRs that provided a summary of intervention assessments, regardless of their duration and follow-up point, were considered eligible for inclusion.

### Study selection and data extraction

EndNote X9 (Clarivate Analytics, Spring Garden, Pennsylvania, USA) software managed search results and deduplication. Two reviewers independently screened all potentially relevant studies based on recorded titles and abstracts and then cross-checked them. In the event of disagreement, the study was provisionally included to obtain additional information. After an initial selection decision was made, the full texts of the chosen studies were downloaded for further review. Two independent reviewers then conducted a new selection process based on a full-text analysis of eligible SRs. An all-researcher meeting was convened to reach a definitive decision on disparate related studies. We extracted information regarding the population, intervention, comparison, and outcomes (PICO) process, certainty of evidence statements, and the number of studies included in each SR.

### Quality assessment

#### Methodological quality assessment

Two independent reviewers used the Assessment of Multiple Systematic Reviews 2 (AMSTAR-2) tool to evaluate the methodological quality of the included SRs. Disagreements were resolved by mutual discussion with a third reviewer until a consensus was reached. AMSTAR-2 consists of 16 items [19], each of which was evaluated as “Yes”, “Partial yes”, or “No”, and overall methodological quality according to the weaknesses in critical domains (items 2, 4, 7, 9, 11, 13 and 15) was categorized as high, moderate, low, or critically low. In other words, there were four categories in the overall assessment results of SRs: “High” was defined as no or one non-critical weakness; “Moderate” meant more than one non-critical weakness; “Low” was one critical flaw with or without non-critical weaknesses; and “Critically low” was defined as more than one critical flaw with or without non-critical weaknesses.

#### Evidence quality assessment of outcomes

Assessment of the evidence quality used the Grading of Recommendations Assessment, Development and Evaluation (GRADE) system was carried out by two reviewers back-to-back. In case of disagreement, two reviewers settled it through discussion. If RCTs were included in SRs, initial confidence in the result would be high. In contrast, the evidence quality for observational studies (OS) was low confidence. Then, three upgrade factors, including larger effects, dose–response gradients, and plausible confounding, as well as five degradation factors, including inconsistency, risk of bias, indirectness, imprecision, and publication bias, were considered. Overall evidence quality was categorized as “High”, “Moderate”, “Low”, or “Very low” [20].

### Data synthesis and analysis

The included SRs were classified according to the topic of the investigation. If multiple SRs on similar clinical topics were identified, we chose the most relevant and best-performed SR for each topic based on the results of the GRADE assessment. Besides, SRs for each topic were depicted only once on the bubble plot. The results of the evidence mapping were presented using characteristic tables of the included SRs and a bubble plot display. Each bubble in the graph represents the evidence evaluated by the SRs investigating the efficacy of ECMO for clinical topics. The visual representation or evidence mapping displays information on four dimensions using a bubble plot: *X*-axis, *Y*-axis, bubble size, and color. This enabled us to provide the following forms of information regarding each included SR.

#### *X*-axis: effect estimate

The mapping presented depended on the certainty of the evidence statement, as reported in each SRs [21]. The “beneficial” denoted that conclusions and results reported apparent beneficial effects without any major concerns regarding supporting evidence. The “probably beneficial” effect indicated that the conclusions did not assert an actual benefit despite a positive treatment effect being reported, or the conclusions reported a potential benefit despite the result showing no significant difference. The “no effect” showed that the conclusions and results provided evidence of no differences between intervention and comparison. The “inconclusive” indicated that the study results were insufficient for the authors to conclude whether the intervention had a definitive or potential effect. The “harmful” implied that the conclusions and results were reported to be a harmful effect. In addition, the primary evaluation criteria encompassed long-term prognosis, clinical symptom outcomes, laboratory inflammation indicators, description of adverse events, and quality of life.

#### Y-axis: literature size estimate

The literature size was defined as the number of primary research studies in the selected SR.

#### Bubble size: numbers of included SRs

The bubble size was used to represent the number of SRs on this topic.

#### Color: evidence quality of the findings

The results of the GRADE system assessment were used to determine confidence, which was divided into four categories: green circles represent “high” evidence quality, blue circles symbolize “moderate”, yellow circles convey “low”, along with red circles reflect “very low”.

## Results

### Literature selection

**Fig. 1:**
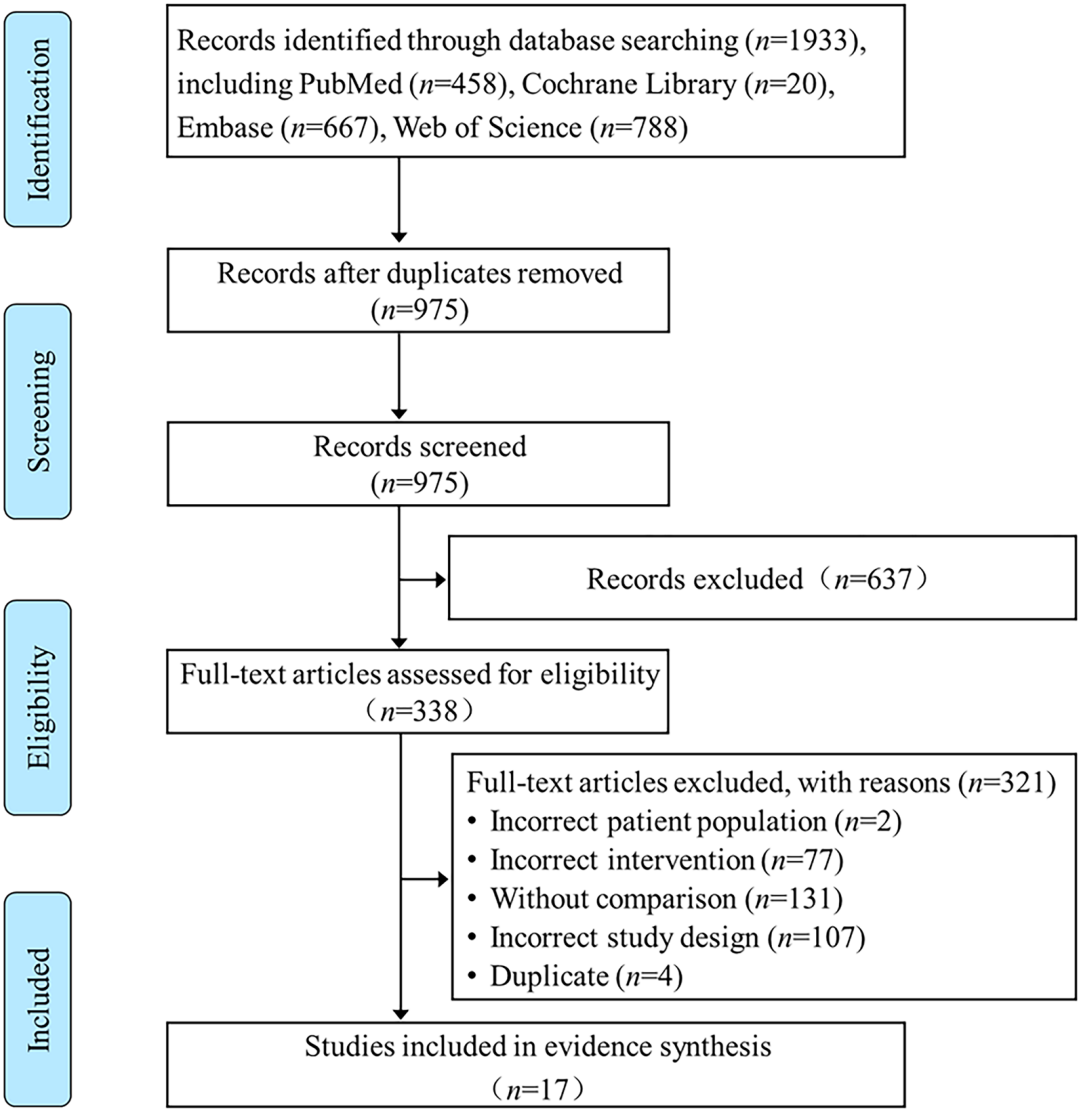
Flow diagram of the literature reviewing process and results

Four electronic databases searches yielded a total of 1933 records from inception to January 2023. After removing duplicates, 975 records were screened based on their titles and abstracts. The initial screening identified 338 potentially relevant studies evaluated against eligibility criteria. A total of 321 articles were deemed ineligible after thoroughly examining of their full text due to non-compliance with the established eligibility criteria. Ultimately, a total of 17 SRs with or without a meta-analysis on ECMO were included for systematic scoping review and evidence synthesis [22–38]. Figure 1 displays all comprehensive review processes, exclusion numbers, and reasons for full-text exclusions.

### Characteristics of included SRs

#### Annual trends in publications

**Fig. 2:**
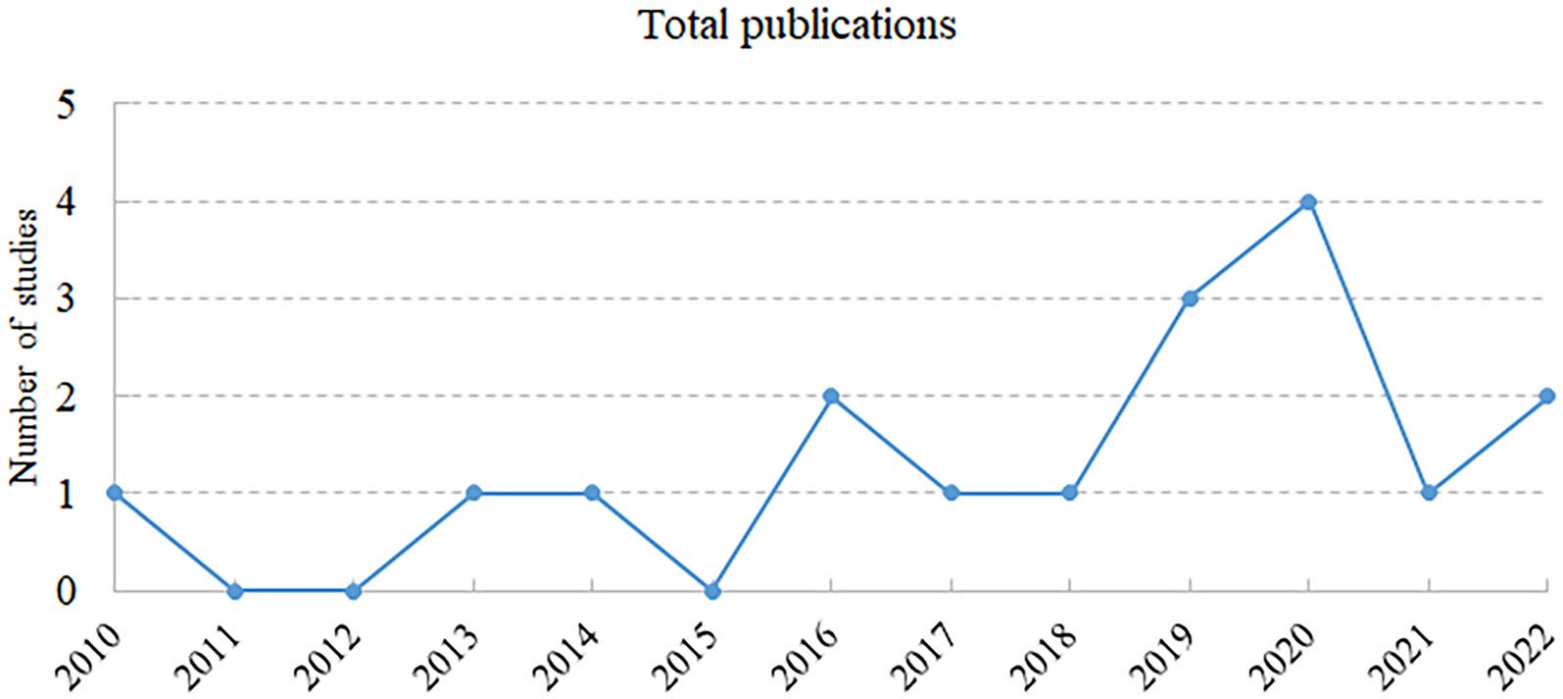
Annual trends in publications of SRs on ECMO

The correlation between the number of studies and the year of publication was plotted to visualize the trend of ECMO studies published over time. SRs on ECMO first appeared in 2010, although our search began with databases construction. Nonetheless, there has been a worldwide increase in ECMO literature, with a peak in 2020, that likely due to the COVID-19 pandemic. Figure 2 depicts the trend in publication of research studies.

#### Geographical distribution

SRs exhibited variation in their country of origin, with the greatest frequency being observed for Canada [24, 27, 28, 31, 34] (*n* = 5). Other nations represented were the United States [22, 33, 35, 38] (*n* = 4), Italy [23, 37] (*n* = 2), China [36] (*n* = 1), Brazil [30] (*n* = 1), France [32] (*n* = 1), Korea [25] (*n* = 1), Netherlands [26] (*n* = 1), and United Kingdom [29] (*n* = 1).

#### Primary studies and participants

The number of studies included for ECMO ranged from 2 to 75, with an average of 14. Ten SRs contained fewer than 10 ECMO-related primary studies, five SRs included 10 to 25 primary studies, and two SRs contained more than 25 primary studies. Three SRs only included the type of randomized controlled trial (RCT), seven SRs included RCT and other study types, and seven SRs were limited to study categories other than RCT. The range of participants included in SRs was 429 to 38,160, averaging 5168. The majority of SRs (*n* = 12) included more than 1000 participants. There was a wide variety of clinical topics in the included SRs, including acute respiratory distress syndrome (ARDS) [31] (*n* = 1), dependent ARDS [38] (*n* = 1), severe ARDS [27, 30, 32, 36] (*n* = 4), acute respiratory failure (ARF) [24] (*n* = 1), ARF due to H1N1 influenza pandemic [22] (*n* = 1), acute lung injury (ALI) due to H1N1 influenza infection [23] (*n* = 1), acute liver failure (ALF) or acute on chronic liver failure (ACLF) [34] (*n* = 1), cardiac arrest [26, 28, 29, 33, 35, 37] (*n* = 6), and cardiac arrest of cardiac origin [25] (*n* = 1). The numbers of primary studies and participants are shown in Table 1. Eligibility criteria for SRs varied across studies showed in Table 2.

**Table 1:** Summary of the included SRs of ECMO treatment

First author, publication year	Disease	Design of primary studies	No. of primary studies	No. of included RCTs	No. of participants	Outcome	Conclusion
Munshi 2019 [31]	ARDS	RCT, observational study	5	2	899	60-Day mortality, treatment failure, mortality at longest available follow-up	Probably beneficial
Shrestha 2022 [38]	Dependent ARDS	RCT, retrospective study, prospective observational study, cohort study	12	2	1208	30-Day mortality, 90-day mortality, in-hospital mortality, ICU mortality, length of hospital stays, average ICU length of stay	Inconclusive
Tillmann 2017 [27]	Severe ARDS	RCT, cohort study	26	1	1674	Survival, adverse events	Inconclusive
Mendes Pedro Vitale, 2019 [30]	Severe ARDS	RCT	2	2	429	Mortality, treatment failure, need for renal replacement therapy, ICU lengths of stay, hospital lengths of stay	Probably beneficial
Alain Combes, 2020 [32]	Severe ARDS	RCT	2	2	429	90-Day mortality, 90-day treatment failure, 28-day mortality, 60-day mortality, ICU-free days, hospital-free days, ventilation-free days, vasopressor-free days, RRT-free days, neurological failure-free days	Beneficial
Zhu, 2021 [36]	Severe ARDS	RCT, retrospective or prospective cohort study	7	2	867	90-Day mortality, 30-day mortality, 60-day mortality, hospital mortality, mortality at the longest duration of follow-up, device-related adverse events (pneumothorax, massive bleeding, intracranial bleeding, cardiac arrest, massive stroke and death due to MV or ECMO)	Beneficial
Munshi, 2014 [24]	ARF	RCT, observational study	10	4	1248	In-hospital mortality, ICU length of stay, adverse events (bleeding, barotrauma, and sepsis)	Inconclusive
Mitchell, 2010 [22]	ARF due to H1N1 influenza pandemic	RCT, cohort study	6	3	827	Mortality	Inconclusive
Alberto Zangrillo, 2013 [23]	ALI due to H1N1 influenza infection	Observational study	8	0	1357	Mortality	Beneficial
Alshamsi Fayez, 2020 [34]	ALF or ACLF	RCT	25	25	1796	Mortality, hepatic encephalopathy outcome, adverse events (hypotension, bleeding, thrombocytopenia, line infections)	Probably beneficial
Ouweneel Dagmar, 2016 [26]	Cardiac arrest	Cohort study	10	0	3127	30-Day survival rate, 30-day favorable neurological outcome	Beneficial
Beyea, 2018 [28]	Cardiac arrest	Case series, cohort study	75	0	5570	Neurologic status at hospital discharge, survival	Inconclusive
Twohig, 2019 [29]	Cardiac arrest	Retrospective or prospective observational study	9	0	26,030	Survival at hospital discharge or 30 days, neurological function	Probably beneficial
Miraglia, 2020 [33]	Cardiac arrest	Cohort study	6	0	1108	30-Day and long-term favorable neurological outcome, 30-day and long-term survival	Probably beneficial
Miraglia, 2020 [35]	Cardiac arrest	Cohort study, case–control study	6	0	1750	Long-term neurological intact survival	Probably beneficial
Scquizzato, 2022 [37]	Cardiac arrest	RCT, observational study	6	2	1177	Survival with favorable neurological outcome at the longest follow-up available, survival at the longest follow-up available/hospital discharge/30 days, rate of neurological impairments	Beneficial
Ahn Chiwon, 2016 [25]	Cardiac arrest of cardiac origin	Retrospective or prospective cohort study	11	0	38,160	Survival, overall neurologic outcome	Probably beneficial

**Table 2:** Eligibility criteria and method of quality appraisal or risk of bias of the included SRs

First author, publication year	Inclusion criteria	Exclusion criteria	Method of quality appraisal or risk of bias
Munshi, 2019 [31]	① RCT and observational study; ② Refractory hypoxia in adults with ARDS (including treatments such as inhaled nitric oxide or high­frequency oscillation); ③ MV plus venovenous (VV) ECMO compared with conventional MV; ④ Reported mortality at any time	Main focused venoarterial (VA) ECMO studies	The cochrane risk­of­bias (ROB) tool and Newcastle­Ottawa Scale (NOS)
Shrestha, 2022 [38]	① Prospective as well as retrospective observational studies and randomized clinical trials; ② Published after 2000; ③ ARDS patients > 18 years of age; ④ Interventions included ECMO (VV/VA or veno arteriovenous (VAV) compared with conventional treatment of MV or other adjunctive therapies; ⑤ Outcomes involving the mortality rate, clinical improvement and recovery, length of hospital stay, adverse effects of ECMO, mean difference of clinical improvement, and healing	① Comments, editorials, viewpoint articles, systematic reviews, or meta-analyses; ② Non-ARDS patients, less than 18 years of age, or pregnant patients; ③ ECMO used for the management of cases other than ARDS; ④ Not mentioned our outcome of interest	The Cochrane ROB 2.0 tool and the Joanna Briggs Institute (JBI) quality assessment tools
Tillmann, 2017 [27]	① Patients > 16 years with severe ARDS as per the Berlin criteria, or classified as having ARDS as per the 1994 American-European Consensus Conference Definition with a PaO2:FiO2 ratio < 100; ② Intervention group received ECMO; ③ Treatment with low tidal volume MV of 8 cm^3^/kg or less; ④ Reported survival to hospital or ICU discharge	Used ECMO as a pre-specified bridge to lung transplantation	NOS
Mendes Pedro Vitale, 2019 [30]	① RCT; ② Adult patients with ARDS; ③ Used ECMO support plus protective MV compared with protective MV alone	Neonatal, pediatric, and experimental data, as well as case series, observational trials and case reports	The Cochrane ROB tool
Alain Combes, 2020 [32]	① RCT; ② Published after 2000; ③ Patients with ARDS fulfilling the American-European Consensus Conference definition or the Berlin definition for ARDS; ④ VV ECMO in the experimental group and conventional ventilatory management in the control group	Not mentioned	The Cochrane ROB tool
Zhu, 2021 [36]	① Randomized and observational studies; ② Adult populations (age ≥ 18 years old); ③ Comparing ECMO therapy with MV alone in the treatment of severe ARDS; ④ Reported mortality outcomes	① Animal studies or case reports; ② lacked a comparison group; ③ included patients < 18 years	Modified Jadad scores, the Cochrane ROB tool, or NOS
Munshi, 2014 [24]	① ARF patients older than 1 month of age; ② Received ECLS; ③ Compared with patients receiving MV; ④ Reported mortality as an outcome	Not mentioned	The Cochrane ROB tool
Mitchell, 2010 [22]	① Controlled trials or cohort studies; ② Reported on the use of ECMO in influenza patients; ③ Included a minimum of 10 patients in each group; ④ Reported comparisons between patients with ARF managed with and without ECMO; ⑤ Reported mortality rates	Neonatal and pediatric studies (patients under 18 years of age)	A nine-point scale combining elements from Jadad’s and Chalmers’ scales
Alberto Zangrillo, 2013 [23]	① Reported on 10 or more patients; ② With confirmed or suspected H1N1 influenza infection; ③ Receiving ECMO	① Reported on fewer than 10 patients treated with ECMO; ② Duplicate publication	NOS
Alshamsi Fayez, 2020 [34]	① RCT; ② Adults with ALF or ACLF; ③ Intervention with any form of artificial or bio-artificial ECLS; ④ Control group received supportive care not including ECLS; ⑤ All-cause mortality or liver-related mortality, bridging to liver transplant, improvement of HE and adverse events such as hypotension, bleeding, thrombocytopenia, line infection, and citrate toxicity as outcomes	Not mentioned	The Cochrane ROB tool
Ouweneel Dagmar, 2016 [26]	① Diagnosed with either refractory in-hospital or out-of-hospital cardiac arrest or cardiogenic shock after AMI; ② Patients with ECLS support and a control group without ECLS support	① Case reports, non-human studies, pediatric studies, and reviews; ② not reported on survival to discharge, 30-day outcome or 6-month outcome	A modified version of NOS
Beyea, 2018 [28]	① Documented OHCA in adults (age ≥ 16 years); ② Used “ECPR” or equivalent search term, as the intervention; ③ Had either CCPR, defined as either basic life support or advanced cardiovascular life support protocols, or no comparator; ④ Reported hospital outcomes	① Under age 16 years; ② Cardiac arrests of traumatic etiology, or patients suffered in-hospital cardiac arrest including in the emergency department	NOS
Twohig, 2019 [29]	① Observational studies; ② Human adult participants (≥ 17 years old); ③ VA ECMO initiated during cardiac arrest (ECPR); ④ Minimal data outcome of 30-day/hospital mortality reported	① Languages other than English; ② Traumatic cardiac arrest; ③ Comparator not CCPR (only for ECPR vs. CCPR papers); ④ Minimal data outcome not reported or reported at other later time intervals	The Risk of Bias in Non-randomized Studies of Interventions (ROBINS-I) tool
Miraglia, 2020 [33]	① Published in English as full-text articles in indexed journals; ② Used propensity score-matched analysis as part of the study design; ③ Adult participants (≥ 18 years old); ④ Resuscitated from in- and out-of-hospital cardiac arrest; ⑤ Received ECPR; ⑥ Reported neurological outcomes	① Review articles, opinions, letters, case reports, case series, meta-analyses, and studies reported insufficient data; ② Conducted on pregnancy, pediatric populations, presumed pregnancy, or patients with a pulse (eg, cardiogenic shock)	NOS
Miraglia, 2020 [35]	① Employing patient-level randomization or cluster randomization comparing ECPR vs. no ECPR and/or conventional CPR; ② Adults suffering in- or out-of- hospital cardiac arrest, with resuscitation attempted by a bystander or healthcare provider; ③ Compared ECMO using pump-driven VA circuits vs. no ECPR and/or conventional CPR; ④ Long-term neurologically intact survival after in- and out-of- hospital cardiac arrest as the primary outcomes of interest	① Considering in- or out-of- hospital cardiac arrest in pediatrics and pregnancy; ② considering in- or out-of- hospital cardiac arrest due to trauma, hypothermia, and toxic substances, as the core interventions provided by healthcare providers (CPR and early defibrillation)	ROBINS-I tool
Scquizzato, 2022 [37]	① Randomized trials and observational studies reporting propensity score-matched data; ② Comparing adult out-of- hospital cardiac arrest patients treated with ECPR with patients treated with CCPR (i.e., basic and advanced life-support maneuvers)	① Feasibility studies; ② Enrolling less than 20 patients; ③ Not reporting the primary outcome of survival with favorable neurological outcome	The Cochrane ROB 2.0 tool
Ahn Chiwon, 2016 [25]	① Adult patients of cardiac-origin arrest (age 18–75 years); ② Does cardiopulmonary resuscitation with ECMO; ③ Compared to conventional cardiopulmonary resuscitation; ④ Survival rate and neurological outcome as outcomes	① Comments, reviews, case reports, editorials, letters, conference abstracts, meta-analyses, or animal studies; ② Languages other than English; ③ Duplicate studies; ④ Irrelevant populations; ⑤ Inappropriate controls	The Cochrane ROB tool

### Methodological quality of included SRs

**Fig. 3:**
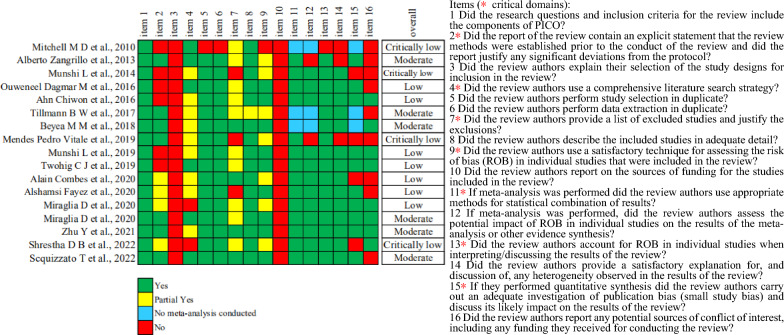
Methodological quality of included SRs

In terms of methodological quality, the overall quality was rated as “Moderate” for six [23, 27, 28, 35–37], seven SRs scored “Low” [25, 26, 29, 31–34], four SRs scored “Critically Low” [22, 24, 30, 38], and “High” for none SRs according to AMSTAR-2 criteria. The most frequent flaws were as follows: lack of a reasonable explanation for the selection of study design type for inclusion, the absence of a report on sources of funding for included studies, a lack of a statement regarding potential sources of conflict of interest, and the absence of a protocol. Figure 3 depicts the methodological quality of the 17 SRs included in the analysis.

### Evidence quality of included SRs

The evidence quality of included one SR was considered high, 5 SRs were moderate and low quality respectively, while 6 SRs were assessed as having very low quality. The results of this evaluation can be found in Table 3.

**Table 3:** Evidence quality of included SRs

Study	Design	Risk of bias	Consistency	Directness	Imprecision	Reporting bias	Strength	Gradient	Confounding	Overall confidence
Munshi, 2019 [31]	RCT, observational study	0	0	0	0	0	0	+ 1	0	⨁⨁⨁◯ moderate
Shrestha, 2022 [38]	RCT, retrospective study, prospective observational study, cohort study	0	− 1	0	0	0	0	0	0	⨁◯◯◯ very low
Tillmann, 2017 [27]	RCT, cohort study	0	0	0	0	0	0	0	0	⨁⨁◯◯ low
Mendes Pedro Vitale, 2019 [30]	RCT	0	0	0	0	-1	0	0	0	⨁⨁⨁◯ moderate
Alain Combes, 2020 [32]	RCT	0	0	0	0	0	0	0	0	⨁⨁⨁⨁ high
Zhu, 2021 [36]	RCT, retrospective or prospective cohort study	0	0	0	0	0	+ 1	0	0	⨁⨁⨁◯ moderate
Munshi, 2014 [24]	RCT, observational study	0	− 1	0	− 1	0	0	0	0	⨁◯◯◯ very low
Mitchell, 2010 [22]	RCT, cohort study	− 1	− 1	0	− 1	0	0	0	0	⨁◯◯◯ very low
Alberto Zangrillo, 2013 [23]	Observational study	0	0	0	0	0	0	0	0	⨁⨁◯◯ low
Alshamsi Fayez, 2020 [34]	RCT	0	0	0	− 1	0	0	0	0	⨁⨁⨁◯ moderate
Ouweneel Dagmar, 2016 [26]	Cohort study	0	0	0	0	0	0	0	0	⨁⨁◯◯ low
Beyea, 2018 [28]	Case series, cohort study	− 1	− 1	0	0	0	+ 1	0	0	⨁◯◯◯ very low
Twohig, 2019 [29]	Retrospective or prospective observational study	0	0	0	0	0	0	0	0	⨁⨁◯◯ low
Miraglia, 2020 [33]	Cohort study	0	0	0	0	0	+ 1	0	0	⨁⨁⨁◯ moderate
Miraglia, 2020 [35]	Cohort study, case–control study	0	0	0	− 1	0	0	0	0	⨁◯◯◯ very low
Scquizzato, 2022 [37]	RCT, observational study	0	0	0	0	0	0	0	0	⨁⨁◯◯ low
Ahn Chiwon, 2016 [25]	Retrospective or prospective cohort study	0	− 1	0	− 1	0	+ 1	0	0	⨁◯◯◯ very low

### Evidence mapping

**Fig. 4:**
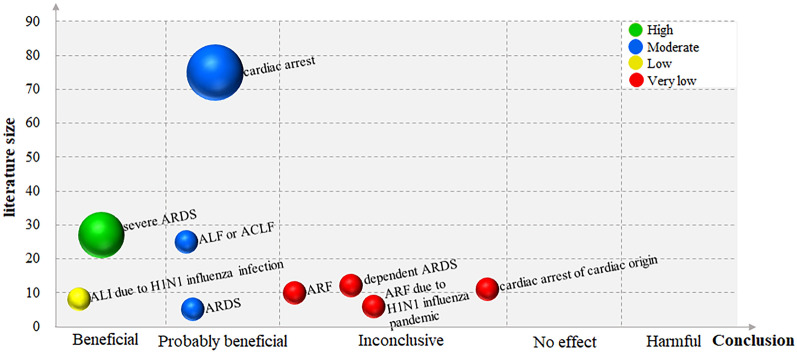
The evidence mapping on ECMO. *X*-axis, effect estimate on the certainty of the evidence statement; *Y*-axis, the number of primary research studies in the selected SR; bubble size, the number of SRs on this topic; bubble color, evidence quality of the findings by GRADE system assessment. *ARDS* adult respiratory distress syndrome, *ALI* acute lung injury, *H1N1* influenza A, *ALF* acute liver failure, *ACLF* acute on chronic liver failure, *ARF* acute respiratory failure

For diseases that overlap, the overall evidence quality was considered. Individual SRs reflected the conclusions, which were confirmed by an internal review. The evidence mapping on ECMO for adults is presented in Fig. 4.

#### Evidence of “beneficial” effect

The effects of ECMO, as indicated by statistically significant pooled treatment effects in SRs, were determined based on a substantial number of research studies that included findings on severe ARDS and ALI due to H1N1 influenza infection.

Four SRs [27, 30, 32, 36] evaluated the effects of ECMO on severe ARDS relative to conventional therapy. Among them, one SR [30] with moderate evidence quality was selected as “probably beneficial” on this mapping, suggestive of the probable efficacy of ECMO in severe ARDS; the efficacy of ECMO and severe ARDS in this study was likely linked to reducing mortality, treatment failure, and the need for renal replacement therapy, but longer ICU and hospital lengths of stay. Two SRs [32, 36], including 2 RCTs with moderate or high evidence quality, were selected as “beneficial” on this mapping, suggesting positive support for ECMO in severe ARDS based on 90-day and 30-day mortality outcomes; additionally, there was no difference in device-related adverse events compared to conventional therapy. The remaining SR [27], with low evidence quality, showed an “inconclusive” conclusion in survival to hospital discharge, indicative of weak confidence to support the effectiveness of ECMO. In general, 75% of SRs (comprising 6 RCTs) were classified under the categories of “beneficial” or “probably beneficial”. The overlapping severe ARDS of four SRs was eventually classified as a “beneficial” conclusion considering the overall evidence quality.

A single SR [23] consisting of eight observational studies, which were of low evidence quality, evaluated the impact of ECMO on ALI due to H1N1 influenza infection compared to conventional therapy. Results indicated that ECMO was feasible and effective in patients with ALI due to H1N1 infection; however, subjects with severe comorbidities or multiorgan failure remained at high risk of in-hospital death if prolonged support (more than one week) was required in the majority of cases, which ended in a “beneficial” conclusion.

#### Evidence of “probably beneficial” effect

A considerable number of research studies on clinical topics such as ARDS, ALF or ACLF, and cardiac arrest were used to determine the promising effects of ECMO, as evidenced by statistically significant pooled effects in SRs.

One SR [31], with moderate evidence quality, compared the effects of ECMO on ARDS to conventional therapy, finding that venovenous ECMO in ARDS adults was associated with lower 60-day mortality (relative risk [RR] = 0.73, 95% confidence interval [CI] 0.58–0.92, *P* = 0.008, *I*^2^ = 0%), but also a moderate risk of bleeding.

One SR [34] with moderate evidence quality comparing extracorporeal life support (ECLS) to usual care found that ECLS might decrease mortality (RR = 0.84, [95% CI 0.74–0.96], moderate certainty) and improve hepatic encephalopathy (RR = 0.71, [95% CI 0.60–0.84], low certainty) in patients with ALF or ACLF. The impact of ECLS on hypotension (RR = 1.46, [95% CI 0.98–2.2], low certainty), bleeding (RR = 1.21, [95% CI 0.88–1.66], moderate certainty), thrombocytopenia (RR = 1.62, [95% CI 1.0–2.64], very low certainty) and line infection (RR = 1.92, [95% CI 0.11–33.44], low certainty) was uncertain.

Six SRs on cardiac arrest produced controversial results [26, 28, 29, 33, 35, 37]. Among them, two SRs [26, 37], both low evidence quality, were selected as “beneficial” on this mapping, suggestive of positive support for ECMO in cardiac arrest based on a 30-day survival rate ([95% CI 6–20%], *P* < 0.001), 30-day favorable neurological outcome ([95% CI 7–20%, *P* < 0.0001), survival with the favorable neurological outcome at the longest follow-up available (OR = 2.11, [95% CI 1.41–3.15], *P* < 0.001), survival at the longest follow-up available (OR = 1.40, [95% CI 1.05–1.87], *P* = 0.02). Three SRs [29, 33, 35] with low, moderate, or very low evidence quality showed a “probably beneficial” conclusion on this mapping, suggesting the probable efficacy of ECMO in cardiac arrest; the efficacy of ECMO and cardiac arrest in these studies was likely associated with improved survival, 30-day and long-term favorable neurological outcome, and long-term neurologically intact survival. The remaining SR [28] of 63 case series and 12 cohort studies concerning out-of-hospital cardiac arrest (OHCA), with very low evidence quality, demonstrated that although a trend toward improved survival with good neurologic outcome was reported in controlled, low-risk of bias cohort studies, a preponderance of low-quality evidence may ascribe an optimistic effect size of extracorporeal cardiopulmonary resuscitation (ECPR) on survival among OHCA patients, rated as “inconclusive” conclusion. On the whole, 83.3% of SRs were classified into “beneficial” or “probably beneficial” categories. For the overlapping cardiac arrest of six SRs, we ultimately rated it as “probably beneficial” conclusion after considering the overall quality of the evidence.

#### Evidence of “inconclusive” effect

This mapping contained several SRs that provided evidence of the potential inconclusive effect of ECMO in treating clinical topics, including dependent ARDS, ARF, ARF due to the H1N1 influenza pandemic, and cardiac arrest of cardiac origin.

According to one SR [38] with very low evidence quality, 30-day mortality (OR = 0.56, [95% CI 0.37–0.84]) and 90-day mortality (OR = 0.59, [95% CI 0.41–0.85]) were reduced in dependent ARDS patients managed with ECMO. However, ECMO management was associated with a 7.28-day increase in ICU duration of stay (MD = 7.28, [95% CI 2.55–12.02]). In addition, there was no statistically significant difference between ECMO and conventional therapy in terms of in-hospital mortality (OR = 0.75, [95% CI 0.40–1.41]), ICU mortality (OR = 1.00, [95% CI 0.36–2.79]), or hospital duration of stay (MD = 3.92, [95% CI − 6.26 to 14.79]).

One SR [24] with very low evidence quality indicated that ECLS was not associated with a mortality benefit in ARF patients (RR = 1.02, [95% CI 0.79–1.33], *I*^2^ = 77%) and was associated with an increased risk of bleeding (RR = 11.44, [95% CI 3.11–42.06], *I*^2^ = 0%). However, a significant mortality benefit was observed in venovenous ECLS studies of higher quality (RR = 0.62, [95% CI 0.45–0.8], *I*^2^ = 25%).

One SR [22] with very low evidence quality demonstrated that there was insufficient evidence to recommend for the use of ECMO among patients with ARF due to the H1N1 influenza pandemic.

One SR [25] with very low evidence quality indicated that ECPR yielded comparable survival (OR = 2.26, [95% CI 0.45–11.20]) and neurologic outcomes (OR = 3.14, [95% CI 0.66–14.85]) to CCPR in the out-of-hospital cardiac arrest of cardiac origin patients. However, in cases of in-hospital cardiac arrest of cardiac origin patients, ECPR demonstrated a significantly higher survival rate (OR = 2.40, [95% CI 1.44–3.98]) and improved neurologic outcomes (OR = 2.63, [95% CI 1.38–5.02]) compared to CCPR.

#### Evidence of “no effect”

No SR clearly declared that ECMO was harmful to clinical topics.

#### Evidence of “harmful” effect

No SR clearly declared that ECMO was harmful to clinical topics.

## Discussion

Evidence mapping is a relatively new method for summarizing scientific evidence on a specific topic. Despite the absence of a standard definition or agreement on its components or application methods, these types of reviews share certain characteristics [18]. Generally, it involves executing a systematic search across various topics to identify knowledge gaps and/or future research needs. It also presents the findings in an approachable format, such as a visual figure, graph, or searchable database [18]. Even without research retrieval and data extraction, evidence mapping can generate a comprehensive list of priority research issues in a topic area, which has the potential to serve as a foundation for study, policy development, and research funding [39].

### Principal findings

There are several SRs for ECMO based on available evidence, with only 17 SRs centered on various diseases meeting the criteria. Most SRs contain a small number of primary studies, indicating limited evidence for this issue. RCT is the most reliable evidence to evaluate the efficacy and safety of interventions [40]. However, the majority of the main studies used to support the efficacy and safety of ECMO were not RCT, according to the SRs included in this evidence mapping, which might be a phenomenon with significant ethical implications.

SR, an essential component of evidence-based medicine, has become the highest level of evidence as it synthesizes all available evidence on a given topic [41]. However, if the quality and criteria of SR differ widely, the findings of reviews may be exaggerated. Although methodological quality assessment is not a core task of evidence mapping, it has been recommended that any review should include this process to assess the consistency of their conclusions [42]. The AMSTAR-2 tool has been used extensively as an effective method to evaluate the methodological quality of SR [43]. Increasing numbers of SR have been conducted on ECMO, but we used the AMSTAR-2 tool to assess methodological quality to ascertain the validity of their conclusions. Unfortunately, we discovered no “High” methodological quality of SRs, but rather six “Moderate” SRs, seven “Low” SRs, and four “Critically Low” SRs. The most frequent shortcomings were as follows: lack of a reasonable explanation for the selection of study design type for inclusion, the absence of a report on sources of funding for included studies, a lack of a statement regarding potential sources of conflict of interest, and the absence of a protocol, all of which would require the attention of future researchers.

Our evidence mapping emphasizes the areas where SRs have reported “beneficial”, “probably beneficial”, “inconclusive”, “no effect”, or “harmful” effects while simultaneously displaying the research concentration and volume. ECMO was beneficial for some clinical topics, such as severe ARDS and ALI due to H1N1 influenza infection. It is probably beneficial for certain clinical topics, such as ARDS, ALF or ACLF, and cardiac arrest. Conclusions regarding dependent ARDS, ARF, ARF due to the H1N1 influenza pandemic, and cardiac arrest of cardiac origin were inconclusive. Significantly, we found no evidence of a harmful association between ECMO and various clinical topics, which may be due to the fact that few RCTs with negative conclusions have been published [44]. The fact that the efficacy and safety outcome of ECMO in treating severe ARDS is not only concluded as having a “beneficial” effect but also supported by “high” quality evidence, indicating that ECMO is a potentially promising support technique for severe ARDS and is also consistent with the ARDS management guidelines, is particularly noteworthy. According to the formal management guidelines of ARDS [45], severe cases of ARDS with PaO_2_/FiO_2_ < 80 mmHg and/or dangerous MV, despite optimization of ARDS management including high PEEP, neuromuscular blocking agents and prone positioning, should probably be considered for venovenous ECMO. The decision to use ECMO should be evaluated early by contacting an expert center with the strong agreement. Being distinct from severe ARDS, ECMO demonstrated a probably beneficial effect in another SR that did not differentiate the severity of ARDS, suggesting that ECMO may have a more effective therapeutic effect in severe ARDS, which requires further research to confirm. The current estimated mortality rate for ARDS is approximately 30–40%, with severe forms of ARDS having higher mortality rates than mild or moderate forms of ARDS [46]. It is highly promising to assert that using ECMO technology will significantly contribute to reducing mortality rates, especially associated with severe ARDS. The continuous advancements in ECMO technology are expected to achieve such a favorable effect.

An additionally interesting finding was that ECMO outcome indicators tend to focus on survival, mortality, and a favorable neurological outcome. However, none of the studies evaluated the quality of life as an outcome or conducted an economic evaluation. In the past few decades, quality of life has emerged as a significant concept and objective for research and practice in the fields of health and medicine, which can inform clinicians and policymakers about how to prioritize and allocate healthcare resources when assessing the benefits of different treatment options [47]. Similarly, economic evaluation contributes to the most efficient allocation of societal resources [48]. ECMO is an essential technology for critically ill patients with cardiopulmonary failure; consequently, evaluations of the quality of life and economic impact are particularly crucial.

Cardiopulmonary failure is a condition that the majority of critically ill patients may experience, indicating that ECMO may be increasingly required for a prolonged time in the future. According to searched studies, high-quality design research was absent on the adverse effects of long-lasting ECMO use. Four of the 17 included SRs reported potential adverse effects of prolonged ECMO use, including bleeding, barotrauma, sepsis, and circuit-related complications. Specifically, two SRs [24, 31] suggested that ECMO may increase the risk of bleeding in ARDS or ARF patients, and ALI due to H1N1 infection patients with severe comorbidities or multiorgan failure remained at high risk of in-hospital death if prolonged support (over one week) was required in most cases [23]. Having said that, only a few studies on the adverse effects of ECMO were included in this mapping, and most primary studies are not RCTs, so the findings were not entirely accurate. Thus, related RCTs ought to concentrate on developing high-quality studies to evaluate the adverse effects of ECMO.

### Evidence gaps and future directions

This evidence mapping has described the research focus, reported in the existing SRs, and identified the gaps in evidence to identify clinical topics that should be prioritized for future research [49]. However, it can not answer more specific questions, such as the optimal parameter selection, application timing and duration for ECMO in a particular specific health topic. To advance our evidence-based understanding of ECMO, we should acquire additional data on the efficacy and safety of ECMO across and within each clinical condition and patient population using meta-analyses of primary studies. In addition, the large number of clinical topics classified as lacking conclusive evidence calls for additional primary research. In some of the clinical topics included in the category of the inconclusive evidence, additional studies have been published, necessitating an update of the present SRs.

### Strengths and limitations

This evidence mapping, unlike previous ones, provides a comprehensive summary of the current evidence for all categories of clinical topics associated with ECMO without restrictions. Moreover, our study conducted a systematic and exhaustive search of four databases and utilized a relatively dependable study design, SR. Then, we used the AMSTAR-2 tool to assess the methodological quality of inclusion in SRs, and the GRADE system to assess the quality of evidence for inclusion in SR outcomes, visually presenting the results of the existing evidence in the form of a bubble plot based on multiple significant dimensions. In addition, we determine the rating of conclusions based on the description of research results and conclusions, which may avoid the uncertainty caused by policy recommendations determined solely based on research results or conclusions in a certain sense [50], which are not only instructive for future research and important for preventing the waste of academic resources, but also essential for policymakers. Nonetheless, it is important to note that this research does have a few limitations. First, we excluded other study designs (such as RCT, case report, cohort study, or cross-sectional study, etc.), even though the fact that SRs generally could provide the highest quality evidence for decision-making. Second, only four frequent literature databases were searched, but literature from other sources, such as clinical trial registration websites, was not focused on, so literature omission was inevitable. Especially since I was unable to find any information related to ECMO and pregnancy. Third, most of the included SRs were based on observational or cohort studies with poor methodological quality, which might have led to bias and affected the intrinsic authenticity of SR to some extent.

## Conclusions

In conclusion, observational or cohort studies, frequently with small sample sizes, have been the most common types of study to evaluate ECMO. AMSTAR-2 tool rated the methodological quality of the included SRs in this mapping as “Critically Low” to “Moderate”. The most beneficial clinical topics of ECMO therapeutic intervention reported by authors for patients are severe ARDS and ALI due to H1N1 influenza infection. However, ECMO for dependent ARDS, ARF, ARF due to H1N1 influenza pandemic, and cardiac arrest of cardiac origin shows an inconclusive effect. These outcomes emphasize the need for future research on new clinical topics and knowledge gaps in this field. Increased efforts are required to improve the methodology quality and reporting process of SRs on ECMO.

## Data Availability

The data that support the findings of this study are available from the corresponding author upon reasonable request.

## Abbreviations

ECMO: Extracorporeal membrane oxygenation
ARDS: Adult respiratory distress syndrome
ARF: Acute respiratory failure
H1N1: Influenza A
ELSO: Extracorporeal Life Support Organization
SR: Systematic reviews
CCPR: Conventional cardiopulmonary resuscitation
AMSTAR-2: The Assessment of Multiple Systematic Reviews 2
GRADE: The Grading of Recommendations Assessment, Development and Evaluation
OS: Observational study
ALI: Acute lung injury
ALF: Acute liver failure
ACLF: Acute on chronic liver failure
OHCA: Out-of-hospital cardiac arrest
ECPR: Extracorporeal cardiopulmonary resuscitation
ECLS: Extracorporeal life support
NOS: Newcastle­Ottawa Scale
JBI: Joanna Briggs Institute
MV: Conventional mechanical ventilation
ROBINS-I: The Risk of Bias in Non-randomized Studies of Interventions

## References

[1] Kalbhenn J, Zieger B (2022). Bleeding during veno-venous ECMO: prevention and treatment. Front Med (Lausanne).

[2] Gupta AK, Kerr LD, Stretton B, Kovoor JG, Ovenden CD, Hewitt JN, Chan J (2022). Trends in the extracorporeal membrane oxygenation literature: a bibliometric analysis in the COVID-19 era. J Extra Corpor Technol.

[3] Zapol WM, Snider MT, Hill JD, Fallat RJ, Bartlett RH, Edmunds LH, Morris AH, Peirce EN, Thomas AN, Proctor HJ, Drinker PA, Pratt PC, Bagniewski A, Miller RJ (1979). Extracorporeal membrane oxygenation in severe acute respiratory failure. A randomized prospective study. JAMA.

[4] Morris AH, Wallace CJ, Menlove RL, Clemmer TP, Orme JJ, Weaver LK, Dean NC, Thomas F, East TD, Pace NL, Suchyta MR, Beck E, Bombino M, Sittig DF, Bohm S, Hoffmann B, Becks H, Butler S, Pearl J, Rasmusson B (1994). Randomized clinical trial of pressure-controlled inverse ratio ventilation and extracorporeal CO_2_ removal for adult respiratory distress syndrome. Am J Respir Crit Care Med.

[5] Peek GJ, Mugford M, Tiruvoipati R, Wilson A, Allen E, Thalanany MM, Hibbert CL, Truesdale A, Clemens F, Cooper N, Firmin RK, Elbourne D, Cesar TC (2009). Efficacy and economic assessment of conventional ventilatory support versus extracorporeal membrane oxygenation for severe adult respiratory failure (CESAR): a multicentre randomised controlled trial. Lancet.

[6] Davies A, Jones D, Bailey M, Beca J, Bellomo R, Blackwell N, Forrest P, Gattas D, Granger E, Herkes R, Jackson A, McGuinness S, Nair P, Pellegrino V, Pettila V, Plunkett B, Pye R, Torzillo P, Webb S, Wilson M, Ziegenfuss M (2009). Extracorporeal membrane oxygenation for 2009 influenza A(H1N1) acute respiratory distress syndrome. JAMA.

[7] Gerke AK, Tang F, Cavanaugh JE, Doerschug KC, Polgreen PM (2015). Increased trend in extracorporeal membrane oxygenation use by adults in the United States since 2007. BMC Res Notes.

[8] Extracorporeal Life Support Organization Available at: http://www.elso.org, Accessed 14th Jun 2023.

[9] Zahringer J, Schwingshackl L, Movsisyan A, Stratil JM, Capacci S, Steinacker JM, Forberger S, Ahrens W, Kullenberg DGD, Schunemann HJ, Meerpohl JJ (2020). Use of the GRADE approach in health policymaking and evaluation: a scoping review of nutrition and physical activity policies. Implement Sci.

[10] Lu C, Lu T, Ge L, Yang N, Yan P, Yang K (2020). Use of AMSTAR-2 in the methodological assessment of systematic reviews: protocol for a methodological study. Ann Transl Med.

[11] Hetrick SE, Parker AG, Callahan P, Purcell R (2010). Evidence mapping: illustrating an emerging methodology to improve evidence-based practice in youth mental health. J Eval Clin Pract.

[12] Bonetti AF, Della RA, Lucchetta RC, Tonin FS, Fernandez-Llimos F, Pontarolo R (2020). Mapping the characteristics of meta-analyses of pharmacy services: a systematic review. Int J Clin Pharm.

[13] Hoffmann TC, Oxman AD, Ioannidis JP, Moher D, Lasserson TJ, Tovey DI, Stein K, Sutcliffe K, Ravaud P, Altman DG, Perera R, Glasziou P (2017). Enhancing the usability of systematic reviews by improving the consideration and description of interventions. BMJ.

[14] Caldwell DM, Welton NJ, Ades AE (2010). Mixed treatment comparison analysis provides internally coherent treatment effect estimates based on overviews of reviews and can reveal inconsistency. J Clin Epidemiol.

[15] Pieper D, Buechter R, Jerinic P, Eikermann M (2012). Overviews of reviews often have limited rigor: a systematic review. J Clin Epidemiol.

[16] Edwards P, Clarke M, DiGuiseppi C, Pratap S, Roberts I, Wentz R (2002). Identification of randomized controlled trials in systematic reviews: accuracy and reliability of screening records. Stat Med.

[17] Haddaway NR, Bernes C, Jonsson BG, Hedlund K (2016). The benefits of systematic mapping to evidence-based environmental management. Ambio.

[18] Miake-Lye IM, Hempel S, Shanman R, Shekelle PG (2016). What is an evidence map? A systematic review of published evidence maps and their definitions, methods, and products. Syst Rev.

[19] Shea BJ, Reeves BC, Wells G, Thuku M, Hamel C, Moran J, Moher D, Tugwell P, Welch V, Kristjansson E, Henry DA (2017). AMSTAR 2: a critical appraisal tool for systematic reviews that include randomised or non-randomised studies of healthcare interventions, or both. BMJ.

[20] Hultcrantz M, Mustafa RA, Leeflang M, Lavergne V, Estrada-Orozco K, Ansari MT, Izcovich A, Singh J, Chong LY, Rutjes A, Steingart K, Stein A, Sekercioglu N, Arevalo-Rodriguez I, Morgan RL, Guyatt G, Bossuyt P, Langendam MW, Schunemann HJ (2020). Defining ranges for certainty ratings of diagnostic accuracy: a GRADE concept paper. J Clin Epidemiol.

[21] Madera AM, Franco J, Ballesteros M, Sola I, Urrutia CG, Bonfill CX (2019). Evidence mapping and quality assessment of systematic reviews on therapeutic interventions for oral cancer. Cancer Manag Res.

[22] Mitchell MD, Mikkelsen ME, Umscheid CA, Lee I, Fuchs BD, Halpern SD (2010). A systematic review to inform institutional decisions about the use of extracorporeal membrane oxygenation during the H1N1 influenza pandemic. Crit Care Med.

[23] Zangrillo A, Biondi-Zoccai G, Landoni G, Frati G, Patroniti N, Pesenti A, Pappalardo F (2013). Extracorporeal membrane oxygenation (ECMO) in patients with H1N1 influenza infection: a systematic review and meta-analysis including 8 studies and 266 patients receiving ECMO. Crit Care.

[24] Munshi L, Telesnicki T, Walkey A, Fan E (2014). Extracorporeal life support for acute respiratory failure. A systematic review and metaanalysis. Ann Am Thorac Soc..

[25] Ahn C, Kim W, Cho Y, Choi KS, Jang BH, Lim TH (2016). Efficacy of extracorporeal cardiopulmonary resuscitation compared to conventional cardiopulmonary resuscitation for adult cardiac arrest patients: a systematic review and meta-analysis. Sci Rep.

[26] Ouweneel DM, Schotborgh JV, Limpens J, Sjauw KD, Engstrom AE, Lagrand WK, Cherpanath T, Driessen A, de Mol B, Henriques J (2016). Extracorporeal life support during cardiac arrest and cardiogenic shock: a systematic review and meta-analysis. Intensive Care Med.

[27] Tillmann BW, Klingel ML, Iansavichene AE, Ball IM, Nagpal AD (2017). Extracorporeal membrane oxygenation (ECMO) as a treatment strategy for severe acute respiratory distress syndrome (ARDS) in the low tidal volume era: a systematic review. J Crit Care.

[28] Beyea MM, Tillmann BW, Iansavichene AE, Randhawa VK, Van Aarsen K, Nagpal AD (2018). Neurologic outcomes after extracorporeal membrane oxygenation assisted CPR for resuscitation of out-of-hospital cardiac arrest patients: a systematic review. Resuscitation.

[29] Twohig CJ, Singer B, Grier G, Finney SJ (2019). A systematic literature review and meta-analysis of the effectiveness of extracorporeal-CPR versus conventional-CPR for adult patients in cardiac arrest. J Intensive Care Soc.

[30] Mendes PV, Melro L, Li HY, Joelsons D, Zigaib R, Ribeiro J, Besen B, Park M (2019). Extracorporeal membrane oxygenation for severe acute respiratory distress syndrome in adult patients: a systematic review and meta-analysis. Rev Bras Ter Intensiva.

[31] Munshi L, Walkey A, Goligher E, Pham T, Uleryk EM, Fan E (2019). Venovenous extracorporeal membrane oxygenation for acute respiratory distress syndrome: a systematic review and meta-analysis. Lancet Respir Med.

[32] Combes A, Peek GJ, Hajage D, Hardy P, Abrams D, Schmidt M, Dechartres A, Elbourne D (2020). ECMO for severe ARDS: systematic review and individual patient data meta-analysis. Intensive Care Med.

[33] Miraglia D, Miguel LA, Alonso W (2020). Extracorporeal cardiopulmonary resuscitation for in- and out-of-hospital cardiac arrest: systematic review and meta-analysis of propensity score-matched cohort studies. J Am Coll Emerg Phys Open.

[34] Alshamsi F, Alshammari K, Belley-Cote E, Dionne J, Albrahim T, Albudoor B, Ismail M, Al-Judaibi B, Baw B, Subramanian RM, Steadman R, Galusca D, Huang DT, Nanchal R, Al QM, Yuan Y, Alhazzani W (2020). Extracorporeal liver support in patients with liver failure: a systematic review and meta-analysis of randomized trials. Intensive Care Med.

[35] Miraglia D, Miguel LA, Alonso W (2020). Long-term neurologically intact survival after extracorporeal cardiopulmonary resuscitation for in-hospital or out-of-hospital cardiac arrest: a systematic review and meta-analysis. Resusc Plus.

[36] Zhu Y, Zhang M, Zhang R, Ye X, Wei J (2021). Extracorporeal membrane oxygenation versus mechanical ventilation alone in adults with severe acute respiratory distress syndrome: a systematic review and meta-analysis. Int J Clin Pract.

[37] Scquizzato T, Bonaccorso A, Consonni M, Scandroglio AM, Swol J, Landoni G, Zangrillo A (2022). Extracorporeal cardiopulmonary resuscitation for out-of-hospital cardiac arrest: a systematic review and meta-analysis of randomized and propensity score-matched studies. Artif Organs.

[38] Shrestha DB, Sedhai YR, Budhathoki P, Gaire S, Subedi P, Maharjan S, Yuan M, Asija A, Memon W (2022). Extracorporeal membrane oxygenation (ECMO) dependent acute respiratory distress syndrome (ARDS): a systematic review and meta-analysis. Cureus.

[39] Bragge P, Clavisi O, Turner T, Tavender E, Collie A, Gruen RL (2011). The global evidence mapping initiative: scoping research in broad topic areas. BMC Med Res Methodol.

[40] Woodbridge A, Abraham A, Ahn R, Saba S, Korenstein D, Madden E, Keyhani S (2018). A cross-sectional analysis of spin in randomized controlled trials. J Gen Intern Med.

[41] Murad MH, Asi N, Alsawas M, Alahdab F (2016). New evidence pyramid. Evid Based Med.

[42] Zeng X, Zhang Y, Kwong JS, Zhang C, Li S, Sun F, Niu Y, Du L (2015). The methodological quality assessment tools for preclinical and clinical studies, systematic review and meta-analysis, and clinical practice guideline: a systematic review. J Evid Based Med.

[43] Perry R, Whitmarsh A, Leach V, Davies P (2021). A comparison of two assessment tools used in overviews of systematic reviews: ROBIS versus AMSTAR-2. Syst Rev.

[44] Jones CW, Handler L, Crowell KE, Keil LG, Weaver MA, Platts-Mills TF (2013). Non-publication of large randomized clinical trials: cross sectional analysis. BMJ.

[45] Papazian L, Aubron C, Brochard L, Chiche JD, Combes A, Dreyfuss D, Forel JM, Guerin C, Jaber S, Mekontso-Dessap A, Mercat A, Richard JC, Roux D, Vieillard-Baron A, Faure H (2019). Formal guidelines: management of acute respiratory distress syndrome. Ann Intensive Care.

[46] Natt BS, Desai H, Singh N, Poongkunran C, Parthasarathy S, Bime C (2016). Extracorporeal membrane oxygenation for ARDS: national trends in the United States 2008–2012. Respir Care.

[47] Haraldstad K, Wahl A, Andenaes R, Andersen JR, Andersen MH, Beisland E, Borge CR, Engebretsen E, Eisemann M, Halvorsrud L, Hanssen TA, Haugstvedt A, Haugland T, Johansen VA, Larsen MH, Lovereide L, Loyland B, Kvarme LG, Moons P, Norekval TM, Ribu L, Rohde GE, Urstad KH, Helseth S (2019). A systematic review of quality of life research in medicine and health sciences. Qual Life Res.

[48] van Mastrigt GA, Hiligsmann M, Arts JJ, Broos PH, Kleijnen J, Evers SM, Majoie MH (2016). How to prepare a systematic review of economic evaluations for informing evidence-based healthcare decisions: a five-step approach (part 1/3). Expert Rev Pharmacoecon Outcomes Res.

[49] Choi TY, Ang L, Ku B, Jun JH, Lee MS (2021). Evidence map of cupping therapy. J Clin Med.

[50] Li Y, Wei Z, Zhang J, Li R, Li H, Cao L, Hou L, Zhang W, Chen N, Guo K, Li X, Yang K (2021). Wearing masks to reduce the spread of respiratory viruses: a systematic evidence mapping. Ann Transl Med.

